# Prevalence of uncontrolled asthma and its associated factors among adult asthmatic patients attending hospitals in Burao City, Somaliland

**DOI:** 10.3389/fmed.2026.1823549

**Published:** 2026-05-15

**Authors:** Abdilaahi Yusuf Nuh, Guta Kune, Zerihun Kura

**Affiliations:** 1 School of Postgraduate Studies and Research, University of Burao, Burao, Somaliland, Somalia; 2 Department of Epidemiology, Faculty of Public Health, Jimma University, Jimma, Ethiopia

**Keywords:** Burao, medication adherence, prevalence, risk factors, Somaliland, uncontrolled asthma

## Abstract

**Objective:**

This study aims to determine the prevalence of uncontrolled asthma and its associated factors among adult asthmatic patients attending hospitals in Burao, Somaliland, in 2025.

**Method:**

A cross-sectional study was conducted at four hospitals from May 1 to June 15, 2025, among 363 adult asthma patients selected using systematic random sampling. Data were collected using a structured, interviewer-administered questionnaire. Asthma diagnosis was confirmed by spirometry performed within 3 months before enrollment, demonstrating reversible airflow obstruction. Descriptive statistics summarized the sample characteristics. Variables with *p* < 0.25 in bivariable analysis were entered into multivariable logistic regression. Model fit was assessed using the Hosmer-Lemeshow test (*p* = 0.423), and multicollinearity was checked using the variance inflation factor (VIF < 2 for all variables). A *p*-value < 0.05 was considered statistically significant.

**Result:**

Out of 382 adult asthma patients approached, 363 were included in the study, yielding a response rate of 98.4%. The mean age of participants was 47.6 ± 9.98 years, with 56.8% being male. Uncontrolled asthma was identified in 57.9% of the participants. Multivariable analysis revealed significant associations with poor medication adherence (AOR = 27.89; 95% CI: 7.75–100.30; *p* < 0.001), persistent asthma severity (AOR = 3.42; 95% CI: 1.27–9.23; *p* = 0.015), a family history of asthma (AOR = 27.51; 95% CI: 2.51–301.60; *p* < 0.001), biomass fuel use (AOR = 8.56; 95% CI: 3.08–23.78; *p* < 0.001), and occupational dust exposure (AOR = 68.65; 95% CI: 3.80–1239.19; *p* = 0.004). Due to small cell counts for dust exposure (*n* = 40 exposed) and family history (*n* = 113 with family history, including only 1 well-controlled case), these estimates have wide confidence intervals and should be interpreted cautiously. Exact logistic regression was performed as a sensitivity analysis, which confirmed the direction and statistical significance of these associations. The presence of air conditioning was also associated with increased odds (AOR = 6.26; 95% CI: 1.44–27.21; *p* = 0.014). Good asthma knowledge approached significance as a protective factor (AOR = 0.45; 95% CI: 0.20–1.04; *p* = 0.060).

**Conclusion:**

The burden of uncontrolled asthma in Burao City is high, with modifiable factors such as medication adherence and follow-up care playing a vital role. Targeted interventions addressing these factors are crucial to improving asthma control in this setting.

## Introduction

Asthma is a common, chronic disorder of the airways that is complex and characterized by variable and recurring symptoms, airflow obstruction, bronchial hyperresponsiveness, and underlying inflammation (1). Every year, about 250,000 people die from asthma, which accounts for 1.1% of disability-adjusted life years (DALY) (2, 3). It has been documented that if not controlled properly, asthma can affect daily activities and lead to significant physical and socio-economic burden with health-related costs (4).

Uncontrolled asthma is characterized by poor symptom control (frequent symptoms or reliever use, activity limited by asthma, nocturnal awakening due to asthma) or frequent exacerbations (≥2/year) requiring Oral Corticosteroids (OCS), or serious exacerbations (≥1/year) requiring hospitalization (5). Patients with uncontrolled asthma use the health care service frequently, resulting in significant productivity loss. Compared to those with well-controlled asthma, people with uncontrolled asthma bear the largest disease burden and financial burden (6).

Uncontrolled asthma leads to increased morbidity, increased utilization of healthcare, and reduced quality of life; thus, it is an important area of inquiry (7, 8). A survey in the U. S. A. 62% of adults with asthma reported having uncontrolled asthma (9). Similarly, an investigation by a study from Saudi Arabia found that uncontrolled asthma had resulted in 45.8% of frequent absenteeism from their condition and therefore, it affects the daily function and quality of life (10). The prevalence is reported as high in several studies conducted in low- and middle-income countries. Among Sudanese asthma patients, 84.5% had uncontrolled asthma (8); in Ethiopia, it was 66.1% (11). The prevalence of uncontrolled asthma has been reported in studies with different settings and populations. For instance, the prevalence was reportedly low at 39.8% in primary care clinics from Saudi Arabia, whereas a meta-analysis study in Ethiopia reported a pooled prevalence of 71.67% (12, 13).

Uncontrolled asthma impairs an individual’s physical activities, emotional life, and social interactions, thus reducing productivity and quality of life and work (14, 15). Numerous factors associated with uncontrolled asthma have included poor medication adherence was a factor seen in various studies, apart from poor follow-up, inadequate inhaler technique, and the presence of comorbidities (8, 11, 12). Other factors as described by multiple studies are low educational level, smoking, obesity, and psychological distress associated with uncontrolled asthma (9, 16). Also, precipitating factors for poorly controlled asthma include a lack of awareness/comprehension of guidelines on asthma and a lack of infrastructure and diagnostic capabilities (17).

Asthma is Somaliland’s fourth most prevalent non-communicable disease, as indicated by the Somaliland Demographic Health Survey 2020 (18). However, data are scarce regarding the prevalence and management of asthma in Somaliland.

Despite the critical gap in local data, no studies to date have investigated the prevalence of uncontrolled asthma or its associated factors in Burao City, Somaliland. Existing evidence from comparable low-resource settings highlights poor treatment adherence, environmental triggers, and medication inaccessibility as key drivers of poor asthma control (19, 20). However, whether these factors or other region-specific determinants predominate in Somaliland remains unexplored, underscoring the urgency of context-specific research to guide public health interventions.

There is an existence of a necessity to address this critical public health gap resulting from a lack of evidence-based studies and to contribute to the improvement of asthma management in Somaliland by identifying the prevalence of uncontrolled asthma and the factors associated with poor asthma control among adult asthmatic patients in Burao City. Therefore, this study aims to determine the prevalence of uncontrolled asthma and factors associated with poor asthma control among adult asthmatic patients, which are essential for improving asthma management.

## Materials and methods

### Study area, design, and period

The study was conducted in four hospitals (Burao General Hospital, Al-Khalifa Hospital, Manhal Specialty Hospital, and Togdheer Private Hospital) in Burao, Somaliland. BGH is the First major referral hospital in the eastern Somaliland region. They offer both inpatient and outpatient services. Burao City is the second largest city of Somaliland and the capital of the Togdheer region, 260 km away from Hargeisa, the capital of Somaliland, and lies at a Latitude of 9^0^ N and a Longitude of 45°E. The climate is semi-arid, and its total population is about 483,724 (21).

Regarding health facilities, the city has eight hospitals (two public and six private), three health centers, and over 12 private clinics. The total number of asthmatic patients in follow-up in these selected health facilities was 419.

An institutional-based cross-sectional study design was employed, and the study was conducted from May 1 to June 15, 2025.

### Study population

All asthmatic patients aged 18 years and older who had a spirometry-confirmed asthma diagnosis within the previous 3 months and were taking anti-asthmatic medications for at least 3 months were included in the study. The 3-month medication duration ensured a stable diagnosis and avoided including patients with transient wheezing or bronchiolitis (22).

The spirometry confirmation required a documented forced expiratory volume in one second (FEV₁)/forced vital capacity (FVC) ratio < 0.70 and evidence of reversibility (≥12% and ≥200 mL increase in FEV₁ after bronchodilator administration), as per standard diagnostic criteria. Patients who consented to participate were enrolled. Patients with respiratory tract infections other than asthma, lung cancer, congestive heart failure, chronic obstructive pulmonary disease, and interstitial lung disease (due to symptom similarity), and pregnant women (due to the variable effects of pregnancy on asthma), were excluded to maintain a strict focus on asthma.

### Sample size and sampling techniques

The sample size of the study was determined using a single-proportional formula. Considering a previous multi-center study done in Harar and Dire Dawa, Eastern Ethiopia, showed a 67.6% proportion of uncontrolled asthma among adult asthmatic patients (23). With a 95% confidence interval and a margin of error (d) of 5%. By adding a 10% non-response rate, the sample size for the study’s first objective became 371. There are two public hospitals in Burao City (Burao General Hospital and Al-Khalifa Hospital), which were included in the study, and Manhal Specialty Hospital and Togdheer Private Hospital were randomly selected from the private hospitals. A systematic random sampling technique was used, with consecutive enrollment of patients aged 18 years and older visiting these facilities during scheduled appointments. The total sample required for the study is proportionally allocated to each hospital based on patient load. Specifically, 194 patients were allocated to Burao General Hospital, 86 to Al-Khalifa Hospital, 54 to Manhal Specialty Hospital, and 37 to Togdheer Private Hospital.

### Data collection procedures and tools

Data was collected using a structured interview questionnaire adopted from the relevant studies (8, 11, 24–27). Six nurses who work in the four hospitals were recruited as the data collectors. After one day of training, data were collected via a face-to-face interview questionnaire and a medical record check review. Asthma control was assessed using a validated tool, the Asthma Control Test (ACT) (28). The ACT is a self-completed questionnaire comprising five questions that determine activity limitation, shortness of breath, nighttime symptoms, use of rescue medication, and patient rating of asthma control over the previous 4 weeks. The questions are scored from 1 (worst) to 5 (best), and the ACT score is the sum of the responses, with a maximum best score of 25. A score of ≥20 is the optimal cutoff point of well-controlled asthma over the previous 4 weeks (28). Uncontrolled asthma was categorized as yes or no, where an ACT score of <20 is considered yes and an ACT score of≥20 is considered no. Spirometry confirmation was verified from patients’ medical records. All included patients had documented evidence of reversible airflow obstruction (≥12% and ≥200 mL increase in FEV₁ after bronchodilator) within the 3 months preceding the study.

Asthma severity was classified according to the National Asthma Education and Prevention Program (NAEPP) Expert Panel Report 3, which relies on symptom frequency, nighttime awakenings, and FEV₁ percentage. However, due to the unavailability of spirometry in the study setting, the classification was adapted using clinical criteria only (symptom frequency, nighttime awakenings, and interference with daily activities), without FEV₁ measurements. The authors acknowledge this as a limitation. The severity categories were: intermittent, mild persistent, moderate persistent, and severe persistent. For analysis, moderate persistent and severe persistent were combined into a single “persistent asthma” category due to small numbers in severe persistent subgroup (1).

Medication adherence was measured using the Medication Adherence Reporting scale for Asthma (MARs-A) (29). To minimize the occurrence of social desirability bias, the questions were phrased as negative statements. On the five-point Likert scale, 1 = always, 2 = often, 3 = sometimes, 4 = rarely, and 5 = never, participants graded their use of medication. Self-reported adherence was represented by the average score of the 10 items’ scores, 1 to 5, where a higher score reflects better adherence. The subjects with a MARs score ≥30 were regarded as showing good adherence to the medication (29–31).

Asthma knowledge was evaluated using consumer questions (CQ) for asthma knowledge. Twelve “true/false” questions of equal weight were included in the questionnaire. Those who scored≥ 9 were categorized as having good knowledge of asthma, and < 9 as having poor knowledge of asthma (32). The attitude section of the questionnaire includes five questions, and the questions were rated on a five-point Likert scale that was used to assess attitude toward asthma. The score ranges from 5–25; patients’ attitudes were categorized into two groups, “positive attitude” for a score ≥ 15 and “negative attitude” for a score < 15 (33).

### Statistical analysis

Data were collected through a structured, interviewer-administered questionnaire developed on the Kobo Toolbox platform. The electronically recorded responses were subsequently exported to STATA version 17 for cleaning and analysis. Descriptive statistics summarized the sample characteristics. For variable selection, all variables with a *p*-value < 0.25 in bivariable analysis were considered candidates for multivariable logistic regression. Multivariable logistic regression analysis was used to identify independent predictors of uncontrolled asthma, reporting adjusted odds ratios (AORs) and 95% confidence intervals (CIs). A *p*-value < 0.05 was considered statistically significant.

Model diagnostics were performed as follows: The Hosmer-Lemeshow goodness-of-fit test was used to assess model calibration (χ^2^ = 8.21, df = 8, *p* = 0.423), indicating adequate model fit. Multicollinearity among independent variables was assessed using the variance inflation factor (VIF), with all VIF values below 2, confirming no significant multicollinearity. The events per variable (EPV) was calculated as 210 events / 12 variables = 17.5, which exceeds the recommended minimum of 10, indicating adequate statistical power. Cell counts for variables with wide confidence intervals (family history of asthma, dust exposure) were reviewed: for family history, 113 participants (31.1%) had a positive history, of whom 112 (99.1%) had uncontrolled asthma and only 1 (0.9%) had controlled asthma; for dust exposure, 40 participants (11.0%) were exposed, of whom 39 (97.5%) had uncontrolled asthma. Due to these small cell counts in the reference category, exact logistic regression using Firth’s penalized likelihood method was performed as a sensitivity analysis, which confirmed the direction and statistical significance of these associations.

### Operational definitions

#### Uncontrolled asthma

Asthma was said to be uncontrolled when the ACT score is <20 (29–31).

#### Medical adherence

The patient was said to have good adherence to the medication when a MARs-A score ≥30 (29–31).

#### 2.6.3 Asthma severity

Graded into four levels according to the National Asthma Education and Prevention Program (NAEPP) Expert Panel Report 3, using both clinical criteria (symptom frequency, nighttime awakenings, activity limitation) and spirometry parameters (FEV₁ percentage predicted) obtained from medical records within the previous 3 months (1).

(a) *Intermittent*: symptoms ≤2 days/week, nighttime awakenings ≤2/month, FEV₁ > 80% predicted, and no interference with daily activities.
(b) *Mild persistent*: symptoms >2 days/week but not daily, nighttime awakenings 3-4/month, FEV₁ ≥ 80% predicted, and minor interference with daily activities.
(c) *Moderate persistent*: symptoms daily, nighttime awakenings >1/week, FEV₁ 60–80% predicted, and some limitations in daily activities.
(d) *Severe persistent*: symptoms throughout the day, nighttime awakenings ≥7/week, FEV₁ < 60% predicted, and extreme limitation of activities.

For analysis, moderate, persistent, and severe persistent were combined into a single “persistent asthma” category due to small cell sizes.

#### BMI classification

Overweight: BMI between 25 and 29.9 kg/m^2^, Obesity: BMI ≥ 30 kg/m^2^, Normal weight: 18.5–24.9 kg/m^2^, Underweight: BMI ≤ 18.5 kg/m2 (34).

## Results

### Socio-demographic characteristics of study participants

The study included 363 participants, resulting in a response rate of 97.8%, with a mean age of 47.6 years (± 9.98). Among them, 206 (56.8%) were male and 157 (43.2%) were female. Regarding educational attainment, 91 (25.1%) had no formal education, 119 (32.8%) had completed primary education, and 59 (16.3%) had achieved higher education or above. A significant majority, 315 (86.8%), lived in urban areas (Table 1).

**Table 1:** Socio-demographic characteristics of adult asthmatic patients at four selected hospitals in Burao City, May 1 to June 15, 2025 (*n* = 363).

Variable	Category	Frequency (*n*)	Percentage (%)
Age (in years)	Mean ± SD	47.6 ± 9.98	
	18–29	19	5.2
	30–39	49	13.5
	40–49	143	39.4
	50–59	112	30.9
	≥60	40	11.0
Gender	Male	206	56.8
	Female	157	43.2
Marital status	Single	110	30.3
	Married	181	49.9
	Divorced/Separated	46	12.7
	Widowed	26	7.2
Education level	No formal education	91	25.1
	Primary school	119	32.8
	Secondary school	94	25.9
	Higher education or above	59	16.3
Occupation	Merchant	87	24.0
	Private employee	90	24.8
	Government employee	110	30.3
	Housewife	42	11.6
	Farmer	22	6.1
	Unemployed	12	3.3
Place of residence	Rural	48	13.2
	Urban	315	86.8
Household income (USD/month)	Mean ± SD	148.5 ± 27.5	
	100–129	92	25.3
	130–159	133	36.7
	160–189	99	27.3
	≥190	24	6.7

### Uncontrolled asthma status

**Figure 1:**
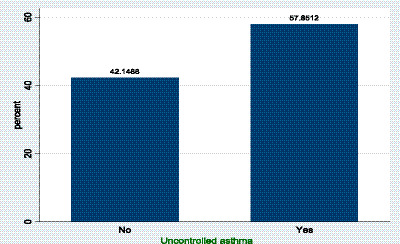
Uncontrolled asthma status of the study participants.

The Asthma Control Test (ACT), a validated self-administered questionnaire, was employed to evaluate asthma control. This instrument comprises five questions that are scored from 1 (worst) to 5 (best), and the ACT score is the sum of the responses, with a maximum best score of 25. A score of ≥20 is the optimal cutoff point of well-controlled asthma over the previous 4 weeks. Based on this criterion, 210 participants (57.9%) were identified as having uncontrolled asthma, while only 153 participants (42.1%) were found to have well-controlled asthma (*Figure 1*).

### Behavioral and knowledge characteristics of the participants

Among the adult asthmatic patients surveyed, a majority, 284 (78.2%), had never engaged in cigarette smoking, while 51 (14.1%) were identified as current smokers, and 28 (7.7%) had a history of smoking. 255 (70.3%) participants reported regular physical exercise, whereas 108 (29.8%) did not partake in regular physical activity. In terms of khat chewing was practiced by 111 (30.6%) of the respondents, while 252 (69.4%) did not engage in this practice. Regarding knowledge about asthma, 220 (60.6%) of the participants demonstrated a good level of knowledge, whereas 143 (39.4%) exhibited poor knowledge. Additionally, 194 (53.4%) displayed a positive attitude toward asthma management, while 169 (46.6%) held a negative attitude (Table 2).

**Table 2:** Behavioral and knowledge characteristics of adult asthmatic patients at four selected hospitals in Burao City, May 1 to June 15, 2025 (*n* = 363).

Variable	Category	Frequency (*n*)	Percentage (%)
Cigarettes smoking	Currently smoking	51	14.05
	Previously smoked	28	7.71
	Never smoked	284	78.24
Regular exercise	No	108	29.75
	Yes	255	70.25
Medical follow-up regularly as scheduled	No	159	43.80
	Yes	204	56.20
Khat	No	252	69.42
	Yes	111	30.58
Knowledge about asthma	Poor knowledge	143	39.39
	Good knowledge	220	60.61
Attitude toward asthma	Positive attitude	194	53.44
	Negative attitude	169	46.56

### Clinical and environmental characteristics of the participant

The mean duration of asthma among participants was 11.61 years (standard deviation [SD] ± 5.02), ranging from a minimum of 2 years to a maximum of 28 years (Table 3). This suggests that most participants have been managing asthma for a considerable period, indicating a chronic disease burden. Regarding body mass index (BMI), 220 (60.6%) were classified as normal body mass index, 106 (29.2%) as low body mass index, and 37 (10.19%) as high body mass index. The most frequently used medications were short-acting beta-agonists (SABA), used by 285 (83.6%), followed by inhaled corticosteroids (ICS) in 159 (46.6%) and oral corticosteroids (OCS) in 115 (33.7%). Combination therapies, such as inhaled corticosteroids (ICS) with long-acting beta-agonists (LABA), were used by 76 (22.3%), and triple therapy (ICS, oral corticosteroids (OCS), and LABA) by 48 (14.1%). Antihistamines were used by 24 (7.0%).

**Table 3:** Clinical and environmental characteristics of adult asthmatic patients at four selected hospitals in Burao City, May 1 to June 15, 2025 (*n* = 363).

Variables	Category	Freq.	Percent (%)
BMI categories	Low BMI (18.5 kg/m^2^)	106	29.2
	Normal BMI (18.5–24.9 kg/m^2^)	220	60.6
	High BM (≥ 25 kg/m^2^)	37	10.19
Duration lived with asthma (Years)	Mean (SD)	—	11.6 (±5.02)
	<5 years	42	11.57
	>5 years	321	88.43
Type of medication	SABA	285	83.6
	ICS	159	46.6
	OCS	115	33.7
	ICS & LABA	76	22.3
	ICS, OCS & LABA	48	14.1
	Antihistamine	24	7.0
Use of biomass for cooking	Yes	148	40.8
	No	215	59.2
Dust/Smoke exposure at work	Yes	40	11.0
	No	323	89.0
Air conditioning at home	Yes	29	8.0
	No	334	92.0
Having pets at home	Yes	101	27.8
	No	262	72.2

**Figure 2:**
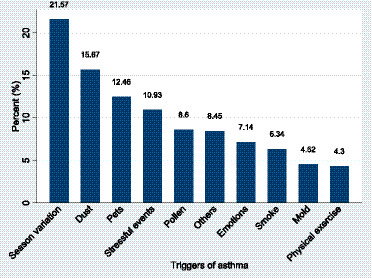
Trigger factors for asthma among the participants.

Environmental exposures were notable; 148 participants (40.8%) used biomass fuel for cooking, and 40 (11.0%) reported occupational exposure to dust or smoke. The majority did not have air conditioning at home (334, 92.0%) and did not keep pets (262, 72.2%). Asthma triggers were diverse, with seasonal variation being the most frequently reported (296, 82.0%), followed by dust exposure (215, 59.6%) and stressful events (150, 41.6%). Other triggers included pollen 118 (32.7%), emotional factors 98 (27.2%), smoke 87 (24.1%), mold 62 (17.2%), physical exercise 59 (16.3%), and other unspecified triggers 116 (32.1%) (Figure 2).

### Factors associated with uncontrolled asthma among the study participants

In the bivariate analysis, several variables exhibited a statistically significant association with uncontrolled asthma (Table 4). Body mass index (BMI) was significantly correlated with asthma control status (χ^2^ = 7.43, *p* = 0.024); notably, 78.38% of high BMI individuals had uncontrolled asthma, compared to 53.77% of low BMI and 55.5% of normal BMI individuals. Similarly, asthma knowledge demonstrated a significant relationship (χ^2^ = 6.45, *p* = 0.011), with 64.1% of those possessing poor knowledge experiencing uncontrolled asthma, compared to 50.9% among those with good knowledge.

**Table 4:** Chi-square test of uncontrolled asthma and associated factors among the asthmatic patients at four selected hospitals in Burao City, May 1 to June 15, 2025 (*n* = 363).

Variable	Category	Uncontrolled asthma		Pearson Chi^2^	*p*-value
		Yes	No		
Gender	Male	119 (57.77%)	87 (42.23%)	0.0014	0.970
	Female	91 (57.96%)	66 (42.04%)		
Marital status	Single	61 (55.45%)	49 (44.55%)	0.5379	0.910
	Married	108 (59.67%)	73 (40.33%)		
	Divorced/Separated	26 (56.52%)	20 (43.48%)		
	Widowed	15 (57.69%)	11 (42.31%)		
Education level	No formal education	56 (61.54%)	35 (38.46%)	1.4822	0.686
	Primary school	71 (59.66%)	48 (40.34%)		
	Secondary school	51 (54.26%)	43 (45.74%)		
	Higher education	32 (54.24%)	27 (45.76%)		
Occupation	Merchant	50 (57.47%)	37 (42.53%)	4.8121	0.439
	Private employee	45 (50.00%)	45 (50.00%)		
	Government employee	65 (59.09%)	45 (40.91%)		
	Housewife	29 (69.05%)	13 (30.95%)		
	Farmer	14 (63.64%)	8 (36.36%)		
	Unemployed	7 (58.33%)	5 (41.67%)		
Residence	Urban	26 (54.17%)	22 (45.83%)	0.3080	0.579
	Rural	184 (58.41%)	131 (41.59%)		
BMI category	Low BMI^a^	57 (53.77%)	49 (46.23%)	7.4327	**0.024**
	Normal BMI	123 (55.91%)	97 (44.09%)		
	High BMI	29 (78.38%)	8 (21.62%)		
Regular exercise	No	49 (45.37%)	59 (54.63%)	0.6544	0.419
	Yes	104 (40.78%)	151 (59.22%)		
Cigarette smoking	Currently smoking	19 (37.25%)	32 (62.75%)	0.5829	0.747
	Previously smoked	12 (42.86%)	16 (57.14%)		
	Never smoked	122 (42.96%)	162 (57.04%)		
Khat	No	108 (42.86%)	144 (57.14%)	0.1696	0.68
	Yes	45 (40.54%)	66 (59.46%)		
Asthma knowledge	Poor knowledge^a^	123 (64.06%)	69 (35.94%)	6.4487	**0.011**
	Good knowledge	87 (50.88%)	84 (49.12%)		
Asthma attitude	Negative	107 (55.15%)	87 (44.85%)	1.2427	0.265
	Positive	103 (60.95%)	66 (39.05%)		
	Yes	150 (42.02%)	207 (57.98%)		
Comorbid chronic illness	No	90 (44.55%)	112 (55.45%)	1.081	0.298
	Yes	63 (39.13%)	98 (60.87%)		
Hospitalized within last 12 months	No	125 (41.81%)	174 (58.19%)	0.0817	0.775
	Yes	28 (43.75%)	36 (56.25%)		
Reason of hospitalization	Asthma^a^	8 (33.33%)	16 (66.67%)	1.6931	0.193
	Others	20 (50.00%)	20 (50.00%)		
Medical follow-up	No^a^	120 (72.29%)	46 (27.71%)	26.1493	**0.000**
	Yes	90 (45.69%)	107 (54.31%)		
Medical adherence	Good^a^	4 (3.51%)	110 (96.49%)	201.276	**0.000**
	Poor	206 (82.73%)	43 (17.27%)		
Exacerbation	No^a^	73 (47.71%)	80 (52.29%)	11.1495	**0.001**
	Yes	137 (65.24%)	73 (34.76%)		
Asthma severity	Mild asthma^a^	106 (42.91%)	141 (57.09%)	68.015	**0.000**
	Persistent asthma	103 (88.79%)	13 (11.21%)		
Family history	No^a^	98 (39.20%)	152 (60.80%)	114.574	**0.000**
	Yes	112 (99.12%)	1 (0.88%)		
Biomass exposure	No^a^	67 (31.16%)	148 (68.84%)	154.039	**0.000**
	Yes	143 (96.62%)	5 (3.38%)		
Dust/Smoke at work	No^a^	164 (51.90%)	152 (48.10%)	35.465	**0.000**
	Yes	46 (97.87%)	1 (2.13%)		
Air conditioning	No^a^	160 (51.78%)	149 (48.22%)	31.40	**0.000**
	Yes	50 (92.59%)	4 (7.41%)		
Pets	No^a^	134 (51.15%)	128 (48.85%)	17.367	**0.000**
	Yes	76 (75.25%)	25 (24.75%)		

^a^Reference category in the multivariate analysis, Significant association *p* < 0.05. Bold values indicate statistically significant associations at *p* < 0.05.

Also, medical follow-up status showed a strong association (χ^2^ = 26.15, *p* < 0.001), with a higher proportion of uncontrolled asthma among patients not receiving regular follow-up (72.3%) compared to those who did (45.7%). Medication adherence emerged as the most significant factor, as 82.7% of patients with poor adherence had uncontrolled asthma versus only 3.5% among those with good adherence (χ^2^ = 201.28, *p* < 0.001). Additionally, recent asthma exacerbations were significantly related to asthma control status (χ^2^ = 11.15, *p* = 0.001), with 65.2% of those who had experienced exacerbations being uncontrolled. Asthma severity was also highly significant (χ^2^ = 68.02, *p* < 0.001), with uncontrolled asthma reported in 42.91% of those with intermittent/mild persistent asthma (combined as “mild cases” for analysis) and 88.79% of those with moderate-to-severe persistent asthma (combined as “persistent cases”).

Environmental and familial factors played a crucial role. A family history of asthma was strongly associated with poor control (χ^2^ = 114.57, *p* < 0.001), as 99.1% of individuals with such a history had uncontrolled asthma, compared to 39.2% of those without a family history. Similarly, biomass fuel exposure (χ^2^ = 154.04, *p* < 0.001), dust/smoke exposure at work (χ^2^ = 35.47, *p* < 0.001), using air conditioning at home (χ^2^ = 31.40, *p* < 0.001), and pet ownership (χ^2^ = 17.37, *p* < 0.001) were all significantly associated with higher rates of uncontrolled asthma.

In the multivariable logistic regression analysis (Table 5). The overall model demonstrated statistical significance, with a likelihood ratio chi-square of 333.72 (*p* < 0.001) and a Pseudo R^2^ value of 0.6634, indicating a robust model fit and that approximately 67.4% of the variability in asthma control status was accounted for by the included predictors.

**Table 5:** Multivariable logistic regression analysis of factors associated with uncontrolled asthma among asthmatic patients at four selected hospitals in Burao City, May 1 to June 15, 2025 (*n* = 363).

Variable	Category	Odds ratio	Std. error	*z*	*p* > |z|	[95% conf. interval]
Asthma knowledge	Poor knowledge	1				0.1955–1.0351
	Good knowledge	0.4499	0.1913	−1.88	0.060	
Bronchial asthma	No	1				1.1471–10.505
	Yes	3.4713	1.9612	2.20	0.028	
Medical follow-up	No	1				0.2427–1.3094
	Yes	0.5637	0.2424	−1.33	0.183	
Medical adherence	Good adherence	1				7.7549–100.2962
	Poor adherence	27.8888	18.2121	5.10	0.000	
Exacerbation	No	1				0.5046–2.8132
	Yes	1.0858	0.4621	0.19	0.847	
Asthma severity	Mild asthma	1				1.2667–9.2328
	Persistent asthma	3.4198	1.7329	2.43	0.015	
Biomass use	No	1				3.0848–23.7793
	Yes	8.5648	4.4623	4.12	0.000	
Dust exposure	No	1				3.8035–1239.187
	Yes	68.6534	101.3405	2.86	0.004	
Air conditioning	No	1				1.4416–27.2082
	Yes	6.2628	4.6936	2.45	0.014	
Having pets	No	1				0.7608–5.7453
	Yes	2.0908	1.0783	1.43	0.153	
Constant (_cons)	—	0.0330	0.0244	−4.61	0.000	0.0077–0.1405

Model summary – LR chi^2^(12) = 333.7, *p* = 0.000; Pseudo R^2^ = 0.6744. _cons represent the baseline odds when all predictors are at reference level, 1- reference category.

Individuals with a comprehensive understanding of asthma were significantly less likely to experience uncontrolled asthma compared to those with limited knowledge (AOR = 0.4499, CI: 0.1955–1.0351, *p* = 0.060). This finding indicates that thorough knowledge of asthma functions as a protective factor, reducing the likelihood of uncontrolled asthma by approximately 57%. Regarding the diagnosis of bronchial asthma, the analysis compared patients with a documented diagnosis of “bronchial asthma” (a specific clinical phenotype characterized by airway hyperresponsiveness) versus those with a non-bronchial asthma diagnosis (e.g., allergic asthma without documented bronchial hyperreactivity). Patients diagnosed with bronchial asthma had 3.47 times higher odds of experiencing uncontrolled asthma compared to those without such a diagnosis (AOR = 3.4713, CI: 1.1471 -- 10.505, *p* = 0.028). This finding suggests that the bronchial asthma phenotype may be associated with more difficult-to-control disease, potentially due to greater airway hyperresponsiveness requiring more intensive management.

Medical adherence emerged as a highly significant predictor. Patients with poor adherence to prescribed asthma medication had 27.89 times higher odds of having uncontrolled asthma compared to those who adhered to their treatment regimen (AOR = 27.8888, 95% CI: 7.7549–100.2962, *p* < 0.001). In terms of asthma severity, individuals diagnosed with persistent asthma exhibited a significantly higher likelihood of experiencing uncontrolled asthma compared to those with mild asthma (AOR = 3.4198, 95% CI: 1.2667–9.2328, *p* = 0.015).

Environmental factors also showed strong and significant associations. Use of biomass fuels for cooking or heating was associated with a markedly increased risk of uncontrolled asthma. Patients exposed to biomass fuel had 8.56 times higher odds of poor asthma control compared to those who did not use such fuels (AOR = 8.5648, 95% CI: 3.0848–23.7793, *p* < 0.001).

Dust exposure showed a very strong association, though with wide confidence intervals (AOR = 68.6534, 95% CI: 3.8035 -- 1239.187, *p* = 0.004). This wide interval reflects the small number of unexposed individuals with uncontrolled asthma (*n* = 1). As a sensitivity analysis, exact logistic regression using Firth’s penalized likelihood method was performed, which confirmed the statistical significance of this association (*p* = 0.002). The large effect size, despite the wide CI, suggests that occupational dust exposure is a clinically meaningful risk factor for poor asthma control in this population. The presence of air conditioning at home was also significantly associated with uncontrolled asthma (AOR = 6.2628, 95% CI: 1.4416 -- 27.2082, *p* = 0.014). This counterintuitive finding is discussed in detail below, with consideration of potential confounding factors and alternative explanations.

## Discussion

This study investigated the prevalence and determinants of uncontrolled asthma among adult patients attending four selected hospitals in Burao City, Somaliland. The study reported a high prevalence of uncontrolled asthma, with 57.9% of the participants exhibiting poor asthma control as determined by the Asthma Control Test (ACT). For instance, studies carried out in Ethiopia documented a 53.3% prevalence of poor asthma control, while Nigeria reported a higher prevalence of 69.3% (24, 35). Such high rates underscore the global challenge of asthma management in low-resource settings, particularly where access to appropriate medications, follow-up services, and patient education is limited (2, 36).

Among the most significant predictors was poor medication adherence, with non-adherent individuals being approximately 28 times more likely to experience uncontrolled asthma (AOR = 27.89, *p* < 0.001). This finding aligns with global evidence that underscores adherence as a fundamental component in achieving asthma control (37, 38). The low adherence rate observed (68.6%) in the current study may be attributed to several factors prevalent in LMICs, such as misconceptions regarding long-term inhaler use, lack of education, inconsistent medication supply, and unaffordable costs (39). Interventions focusing on behavioral counseling, simplified drug regimens, and health literacy have been demonstrated to significantly enhance medication adherence and, consequently, asthma outcomes (40).

In addition to that, asthma severity also showed a strong relationship with control status. Patients with moderate-to-severe persistent asthma (combined as “persistent asthma”) were about 3.42 times more likely to have uncontrolled symptoms than those with intermittent or mild persistent asthma (combined as “mild asthma”) (AOR = 3.42, *p* = 0.015). This finding underscores the importance of continuous healthcare engagement and structured monitoring in the management of chronic diseases. Previous studies have demonstrated that frequent physician follow-up facilitates timely medication adjustments, reinforcement of inhaler techniques, and early management of exacerbating conditions (38, 41).

A family history of asthma emerged as another strong predictor (AOR = 27.51), suggesting possible genetic and shared-environmental contributions to asthma severity and control. This confirms previous research suggesting that individuals with a familial background of asthma might necessitate more intensive management strategies (42). Nevertheless, while genetic factors are influential, their effects can be mitigated through optimal care and environmental control measures.

Environmental exposures were significantly associated with uncontrolled asthma in this population. The use of biomass fuel for cooking was associated with an almost nine folds increase in the likelihood of uncontrolled asthma (AOR = 8.56, *p* < 0.001), while exposure to dust and smoke in occupational settings increased the odds nearly 69-fold (AOR = 68.65, *p* = 0.004), making these among the most potent environmental predictors. These findings are in line with research from similar contexts in Africa and the Middle East, where such exposures significantly impair respiratory health (43, 44). Reducing exposure to indoor pollutants such as biomass smoke through public health initiatives promoting clean energy alternatives is a key strategy endorsed by the WHO and should be a priority in asthma prevention and control in Somaliland.

The presence of air conditioning in homes was unexpectedly associated with increased odds of uncontrolled asthma. Several potential explanations warrant consideration. First, this finding may reflect reverse causality or confounding by socioeconomic status, as air conditioning units are more common in urban, higher-income households where other unmeasured risk factors (e.g., sedentary lifestyle, processed food consumption, or differing healthcare-seeking behaviors) may predominate. Second, poorly maintained air conditioning systems can accumulate dust, mold, and other allergens that are recirculated indoors, potentially exacerbating respiratory symptoms. Third, the thermal shock from moving between air-conditioned indoor environments and the hot, arid outdoor climate of Burao City may trigger bronchoconstriction in susceptible individuals. Fourth, given the small number of participants with air conditioning (*n* = 29, 8.0%), this finding may be unstable; sensitivity analysis excluding this variable did not materially change other effect estimates. The authors recommend cautious interpretation of this finding and emphasize the need for further research to explore the mechanisms underlying this association before any public health recommendations are made.

Also, the level of patient knowledge also approached statistical significance (*p* = 0.06), suggesting a trend toward improved control among patients with substantial asthma knowledge. Previous studies conducted in Ethiopia and Vietnam have demonstrated that patient knowledge significantly influences self-management behaviors, including medication adherence, trigger avoidance, and timely health-seeking (11, 45). Consequently, patient education programs tailored to the local language and context should be integrated into routine asthma care services in Burao.

### Limitations

The authors acknowledge several limitations. First, the cross-sectional design precludes establishing causal relationships. Second, although all participants had spirometry-confirmed asthma from medical records within 3 months before enrollment, repeat spirometry was not performed at data collection, so severity classification relied on historical lung function. Third, self-reported instruments (MARs-A, ACT) are subject to recall and social desirability bias. Fourth, the hospital-based sampling may limit generalizability to all asthmatic patients. Fifth, wide confidence intervals for dust exposure and family history reflect small cell counts (e.g., only one well-controlled case in each exposed group); exact logistic regression confirmed significance, but estimates should be interpreted cautiously. Sixth, the air conditioning finding (*n* = 29 exposed) requires validation due to small numbers and potential confounding. Seventh, the single-city design (Burao) limits generalizability to other regions.

## Conclusion

This study revealed that more than half of adult asthmatic patients in Burao City suffer from uncontrolled asthma, indicating a substantial public health concern. Poor medication adherence, asthma severity, family history of asthma, and environmental exposures such as biomass fuel use and occupational dust were identified as the most significant determinants. The association with air conditioning, while statistically significant, requires further investigation before causal inferences can be drawn. These findings highlight the urgent need for context-specific interventions that strengthen patient education, improve access to medications, promote adherence, and reduce exposure to environmental triggers. Incorporating routine follow-up care and tailored community-based awareness programs will be crucial to reducing the burden of uncontrolled asthma and improving the quality of life for patients in Somaliland.

## Data Availability

The raw data supporting the conclusions of this article will be made available by the authors, without undue reservation.
